# Removal of a Broken Cannulated Femoral Nail: A Novel Retrograde Impaction Technique

**DOI:** 10.1155/2013/601982

**Published:** 2013-11-18

**Authors:** Kongkhet Riansuwan, Direk Tantigate, Banchong Mahaisavariya

**Affiliations:** Department of Orthopaedic Surgery and Rehabilitation, Faculty of Medicine Siriraj Hospital, Mahidol University, Bangkok 10700, Thailand

## Abstract

This report presents a surgical technique to remove a broken cannulated nail from the femur. A Harrington rod was modified for retrograde impaction of the retained fragment. The broken implant was finally removed without complication. This particular procedure was safe, simple, and promising.

## 1. Introduction

Removal of a broken femoral nail especially a retained distal fragment is a challenging procedure in orthopaedic surgery. Generally, pulling of the broken distal fragment is determined as a primary procedure for removal; however, slippage of the extraction device remains a major course of failure. Retrograde impaction is another promising method to overcome such problem. In this particular situation, an appropriate impactor is a key factor of success. Normally, another intramedullary nail or an extraction rod can be applied for such instance [1]. However, the diameter of an impactor should be a major concerning factor regarding knee injury. We, therefore, propose an idea applying a round-end Harrington rod which is smaller than ordinary nail as an impactor for retrograde impaction of the broken cannulated femoral nail.

## 2. A Case Report and Operative Technique

**Figure 1:**
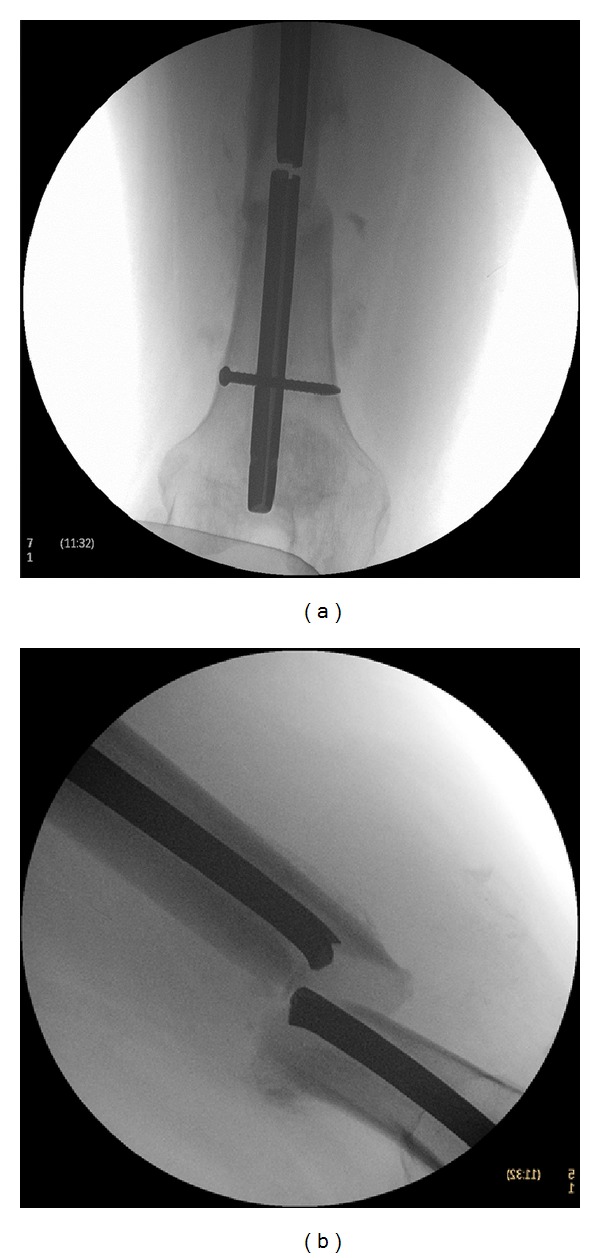
The radiographs show displaced fracture at distal 1/3 of the right femur with a retained broken AO femoral nail in (a) AP and (b) lateral view.

A 35-year-old Thai man underwent closed femoral nailing as a treatment of right femoral shaft fracture. The implant was 11 × 38 mm AO interlocking nail for the femur. Six months later, he sustained another road traffic accident and presented with deformity and pain at the right thigh. The radiographs revealed distal 1/3 femoral shaft fracture with displacement and retained broken femoral nail (Figures 1(a) and 1(b)). Therefore, implant removal and nail exchange by closed technique were determined as a definite treatment. 

**Figure 2:**
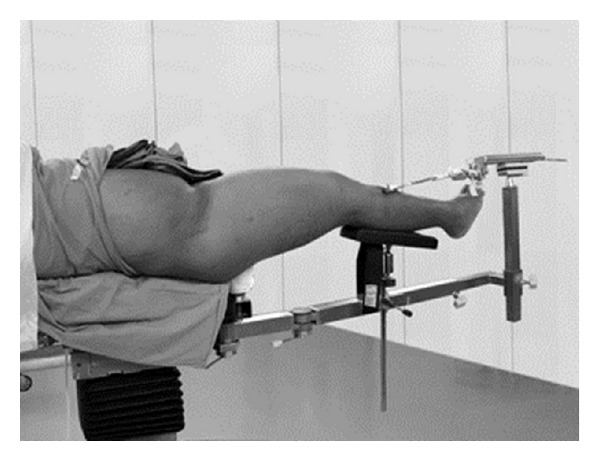
The picture shows patient positioning on a fracture operating table with a skeletal traction applied at the proximal tibia.

**Figure 3:**
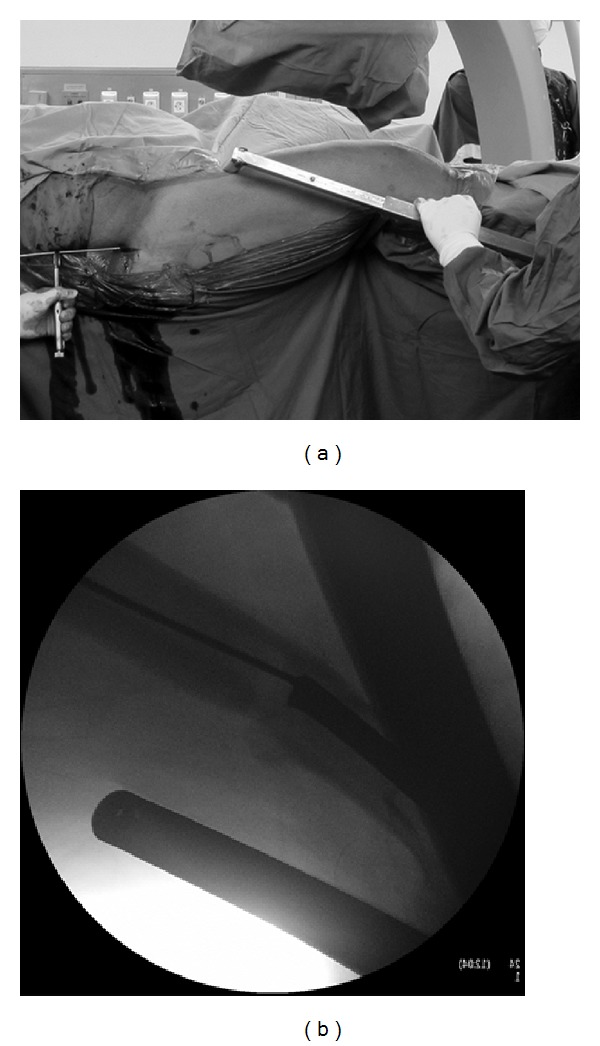
(a) The fracture is closely reduced by using the F-shaped reduction clamp and (b) reduction is maintained by an intramedullary guide wire.

**Figure 4:**
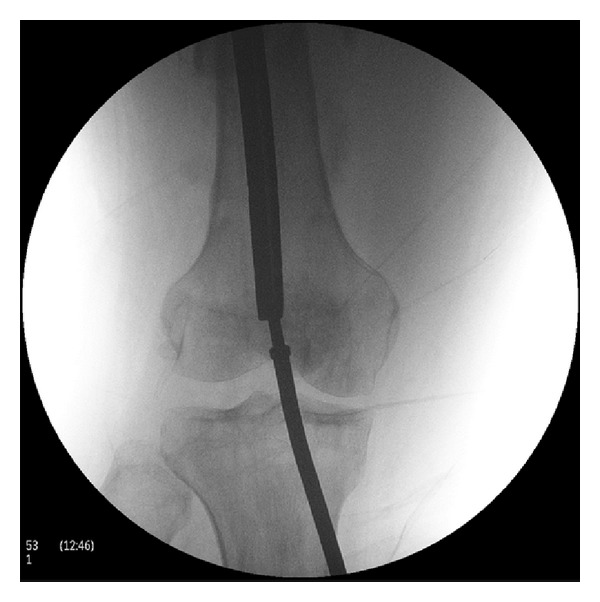
The picture shows application of a Harrington rod through an entry point at the intercondylar notch in order to function as an impactor for nail removal.

**Figure 5:**
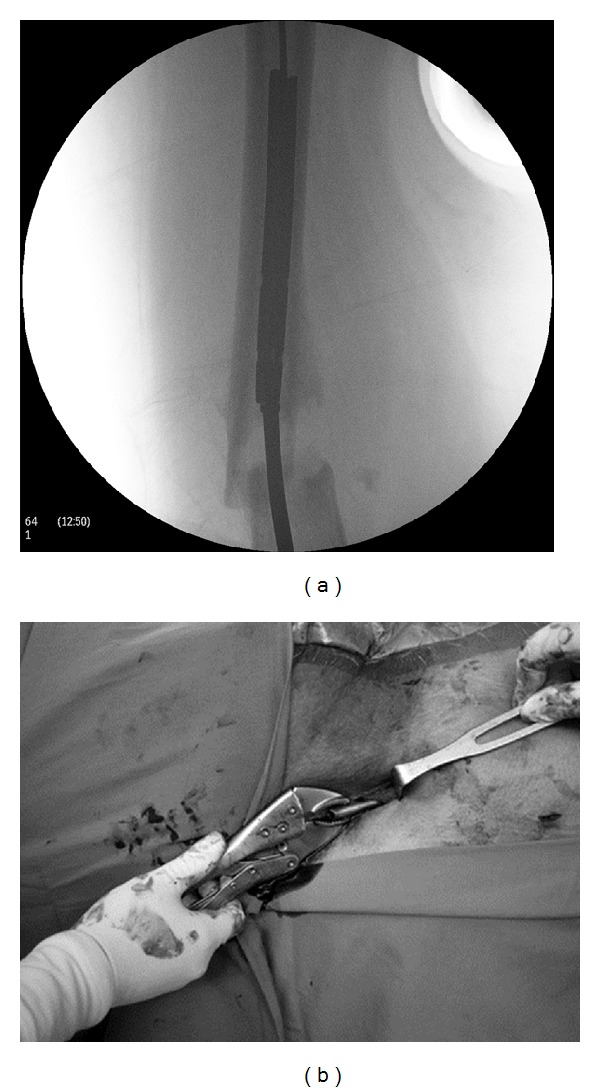
The broken nail is retrogradely impacted and finally removed from the proximal wound.

The patient was placed supine on a fracture operating table with pulling the affected leg on a skeletal traction at the proximal tibia (Figure 2). The proximal part of the nail was removed routinely by using the specific removal equipment. In order to protect the surrounding soft tissue regarding biology of fracture healing, the distal part of the broken nail was determined to be removed by closed technique. The fracture was reduced using the F-shaped reduction clamp (Figure 3(a)). A nail-driving guide wire was inserted into the medullary canal of the femur and passed across the fracture site into the lumen of the distal portion of the broken nail in order to keep fracture alignment and tract of nail removal (Figure 3(b)). Intercondylar notch of the femur was then perforated by an Awl reamer through a small incision and medial parapatellar approach. After enlarging the entry point with a 6 mm T-reamer, a contoured Harington rod (6 × 400 mm) was inserted through the entry point at the femoral condyle until the round tip of the rod engaged into the nail's lumen (Figure 4). Multiple impactions could then be applied on a wisegrip or T-chuck handle gripping the Harrington rod firmly. The nail was impacted upward along the tract controlled by the guide wire and eventually removed directly from the wound (Figures 5(a) and 5(b)). 

## 3. Result

The broken nail could be removed successfully followed by nail exchange without complication. No significant knee pain or functional limitation has been complained.

## 4. Discussion

Broken femoral nail removal is recently not an uncommon procedure in orthopaedic surgery. In general, the proximal portion of a broken nail is routinely removed without difficulty while challenge remains in the part of distal segment removal. For this instance, closed technique has been usually attempted as a primary procedure in order to preserve the surrounding soft tissue. Therefore, many surgical techniques have been published in the literatures to achieve such purpose [2–4]. 

For broken cannulated nails, the distal portion can be removed by pulling or pushing of the retained implant from either the proximal or distal femoral canal. Normally, pulling technique is determined primarily because it is sequential following proximal nail removal and knee arthrotomy is not required. Many instruments such as hook, femoral head cork screw, smaller nail, multiple guide wires, and guide wire with washer have been recommended to be used as an extractor [2, 5–10]. Although such procedure provides a promising outcome, slippage remains the major cause of technical failure. Thus, pushing or impaction is another option to overcome the problem; however, further knee injury is another concern. Unintentional condylar fracture might be at risk for antegrade impaction [11] especially in a small femoral condyle like Asian's condyle. Therefore, retrograde impaction using the same portal as retrograde nailing should minimize the risk of such fracture.

For retrograde impaction, size and length of the impactor remain a concerning issue in order to perform its function and compromise the risk of knee injury. Ordinary bone impactor is too short to be used for such instance. We found that Harrington rod can be perfectly modified as an impactor for this particular condition. Diameter of the rod is just only 6 mm; the round-end tip can engage into the broken nail lumen while the rod shoulder can hit against the nail tip allowing a mechanical benefit during impaction and low risk of slippage. The maximal working length of Harrington rod in our practice, tip-shoulder distance, is 38 mm which is suitable for retrograde impaction of the broken nail at the level of midshaft or distal 1/3. This method is simple, safe and no serious complication occurs in our experience.
